# Establishment of a quantitative RT-PCR detection of SARS-CoV-2 virus

**DOI:** 10.1186/s40001-021-00608-5

**Published:** 2021-12-17

**Authors:** Yushen Jiang, Shanming Zhang, Hong Qin, Shuai Meng, Xuyi Deng, He Lin, Xiaoliang Xin, Yuxin Liang, Bowen Chen, Yan Cui, YiHeng Su, Pei Liang, GuangZhi Zhou, Hongbo Hu

**Affiliations:** 1 Rapid Diagnostics Laboratory, Hangzhou DIAN Medical Laboratories, No. 329 of Jinpeng Street, Xihu District, Hangzhou, 310030 China; 2 Teddy Clinical Research Laboratory (Shanghai) Limited, Shanghai, 200433 China; 3 Jiangxi DIAN-HUAXING Medical Laboratories, NanChang, 330029 China; 4 Tianjin DIAN Medical Laboratories, Tianjin, 300300 China; 5 WuHan DIAN Medical Laboratories, Wuhan, 430034 China

**Keywords:** SARS-CoV-2 virus, COVID-19, RT-PCR, Quantitative, RNA

## Abstract

**Background:**

The outbreak of novel coronavirus disease 2019 (COVID-19) has become a public health emergency of international concern. Quantitative testing of SARS-CoV-2 (severe acute respiratory syndrome coronavirus 2) virus is demanded in evaluating the efficacy of antiviral drugs and vaccines and RT-PCR can be widely deployed in the clinical assay of viral loads. Here, we developed a quantitative RT-PCR method for SARS-CoV-2 virus detection in this study.

**Methods:**

RT-PCR kits targeting E (envelope) gene, N (nucleocapsid) gene and RdRP (RNA-dependent RNA polymerase) gene of SARS-CoV-2 from Roche Diagnostics were evaluated and E gene kit was employed for quantitative detection of COVID-19 virus using Cobas Z480. Viral load was calculated according to the standard curve established by series dilution of an E-gene RNA standard provided by Tib-Molbiol (a division of Roche Diagnostics). Assay performance was evaluated.

**Results:**

The performance of the assay is acceptable with limit of detection (LOD) below 10E1 copies/μL and lower limit of quantification (LLOQ) as 10E2 copies/μL.

**Conclusion:**

A quantitative detection of the COVID-19 virus based on RT-PCR was established.

**Supplementary Information:**

The online version contains supplementary material available at 10.1186/s40001-021-00608-5.

## Introduction

Facing the emergence of coronavirus disease-2019 (COVID-19) worldwide, dozens of RT-PCR virus detection methods targeting SARS-CoV-2 (severe acute respiratory syndrome coronavirus 2) have been established over the past 6 months while few of them are quantitative methods [1–3]. Some researches displayed that clinical manifestations of other coronavirus lung diseases such as severe acute respiratory syndrome (SARS) were related to viral load [4, 5]. Many studies have focused on the SARS-CoV-2 viral load in specimens of infected patients, and high viral loads were detected soon after symptom onset [6–8]. Moreover, dozens of clinical trials targeting SARS-CoV-2 have been run all over the world, viral load determination before and after treatment is critical in evaluating clinical efficacy. Therefore, quantitative testing of the COVID-19 virus is demanded in laboratories worldwide, especially in clinical trials of new treatment. In this study, we developed a quantitative COVID-19 detection method based on the Roche RT-PCR coronavirus E gene detection kit. Its performance is experimentally confirmed and evaluated, and we also employed it in the virus detection of clinical trials LOTUS China (Lopinavir Trial for Suppression of SARS-Cov-2 in China) [9] and NCT04257656 (Remdesivir Trial for Suppression of SARS-Cov-2 in China) [10].

## Materials and methods

### Method design

SARS-CoV-2 virus composed of 29,881 bp is a single-strand RNA virus which is divided into ORF region, S gene, M gene, E gene and N gene [11]. This novel coronavirus detection method is based on the qPCR platform of Roche Diagnostics. Specific primers and probes are designed to detect three genes: RNA-dependent RNA polymerase (RdRP), N gene and E gene.

RNA-dependent RNA polymerase (RdRP) located at the ORF 1ab region is the specific gene that can identify the SARS-CoV-2 virus from COV NL63, 229E, HKU, OC43 or MERS. A fragment of 100 bp in length is designed to the conserved region of the RdRP gene and detected by a new FAM probe labeled for targeting fragment. SARS-CoV-2 virus, SARS virus and bat virus-specific FAM markers were used to detect the amplified fragment of 126 bp in the N gene region and 76 bp in the E gene region. All the primer and probe sequences were designed according to WHO published virus sequence and/or published in the previous paper [10].

Using the E gene region for primary screening of virus infection, the highly conserved N gene can specifically detect the SARS-CoV-2 virus and some others to confirm SARS infection, the RdRp gene is used to identify SARS-CoV-2 virus with high specificity. Medical records between January 10 and January 20, 2020 showed that a 55-year-old male and his wife presented with fever, cough, and shortness of breath, after short-term exposure to Wuhan Huanan seafood market for 2 days. Patients obtained sputum or endotracheal aspirates at admission for identification of possible causative bacteria or fungi. Additionally, chest X-rays or chest CT were given and treated in isolation. Patient were given antiviral treatment for 3–14 days, including oseltamivir (75 mg every 12 h, orally), ganciclovir (0·25 g every 12 h, intravenously), and lopinavir and ritonavir tablets (500 mg twice daily, orally). To the best of our knowledge, there are no identified variant of SARS-CoV-2 between the period of January 10 and January 20, 2020. Therefore, only when the R gene is positive and either N gene or E gene is positive could be regarded as SARS-CoV-2 virus-specific infection.

### Materials for detection

RNA was extracted using the MagNA Pure 96 system (Roche, Germany) with the High Pure Viral RNA Kit (Roche, Germany. Product No. 11858882001).

A LightMix® Modular EAV RNA Extraction Control (580) kit (Roche, Germany. Product No. 07654243001) was used for extraction and reverse-transcription PCR control.

EAV is in vitro transcribed RNA and not viral RNA or a virus provided as a virus extraction control. The control is a 70-bp-long fragment from the Equine Arteritis Virus genome, which is amplified with specific primers and detected with an Atto647-labeled hydrolysis probe.

A LightCycler® Multiplex RNA Virus Master kit (Roche, Germany. Product No. 06754155001) one-step real-time reverse-transcription PCR kit was used for reverse-transcription RT-PCR.

The E-gene RNA single positive control (Tib-Molbiol, Germany. Product No. 30743771) contains 10E10 copies of RNA in one vial. The N-gene RNA single positive control (Tib-Molbiol, Germany. Product No. 30743671) contains 10E10 copies of RNA in one vial. The RdRp-gene RNA single positive control (Tib-Molbiol, Germany. Product No. 30744071) contains 10E10 copies of RNA in one vial.

LightMix® Modular SARS-CoV (COVID19) E-gene kit (Roche, Germany. Product No. 09155368001), LightMix® Modular SARS-CoV (COVID19) N-gene kit (Roche, Germany. Product No. 09155350001), LightMix® Modular SARS-CoV (COVID19) RdRP-gene kit (Roche, Germany. Product No. 09155376001) based on a previously published assay targeting E gene (envelope protein gene), N gene (nucleocapsid protein gene) and RdRP gene (RNA-dependent RNA polymerase gene) of coronavirus are employed for use on Roche cobas® Z480 RT-PCR system [12].

### Pre-experiment

The purpose of the pre-experiment was to evaluate and select suitable genes for viral load quantification. The assay was conducted according to the instruction of the kit. The standards for RdRP, N and E genes were prepared freshly for each batch. One tube of storage solution was thawed and dilution rows from 10E6 to 10E1 copies/μL were obtained by serial 1:10 dilution (10 μL of solution diluted with 90 μL of PCR-grade water).

**Fig. 1:**
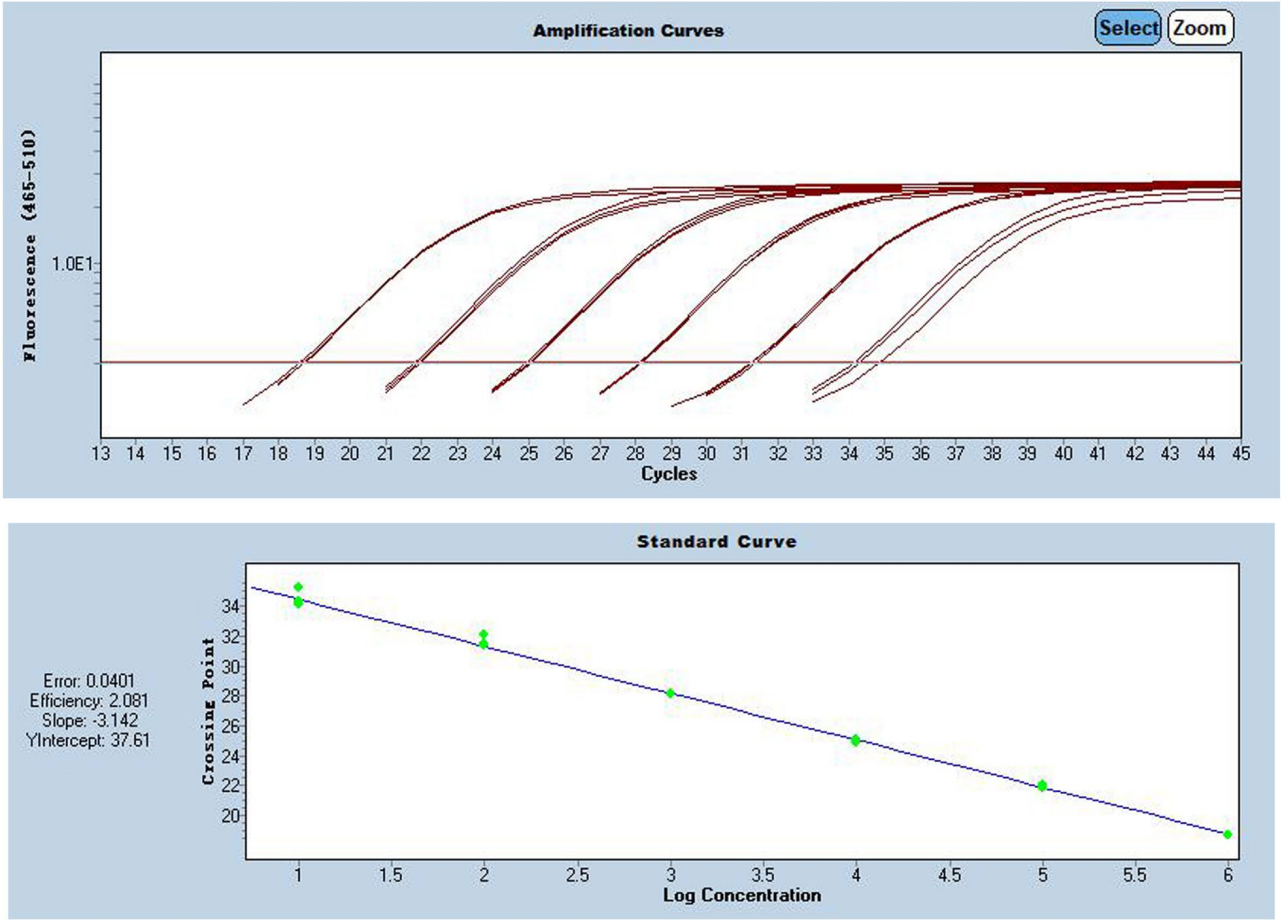
Pre-experiment of E-gene amplification curve

**Fig. 2:**
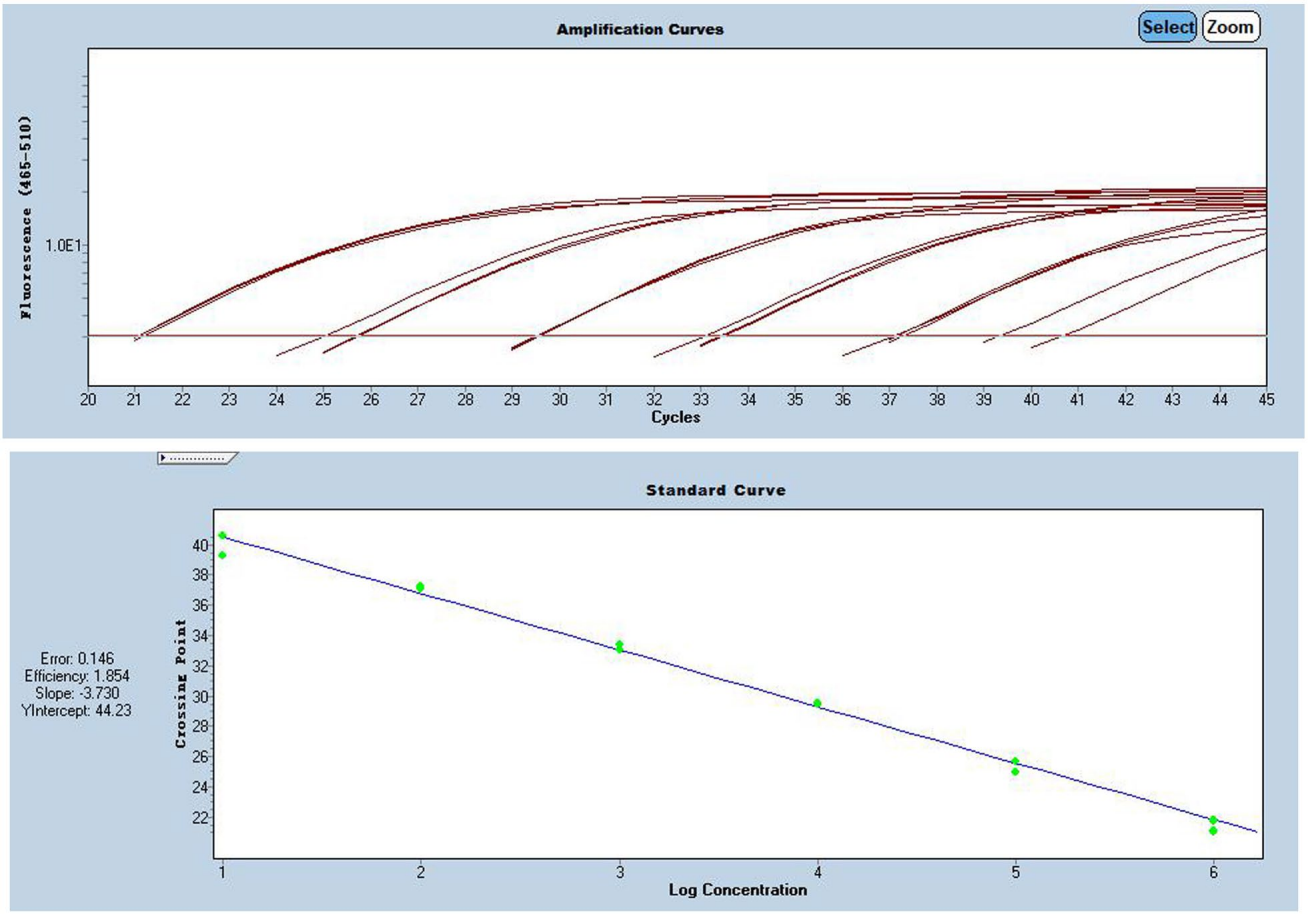
Pre-experiment of N-gene amplification curve

**Fig. 3:**
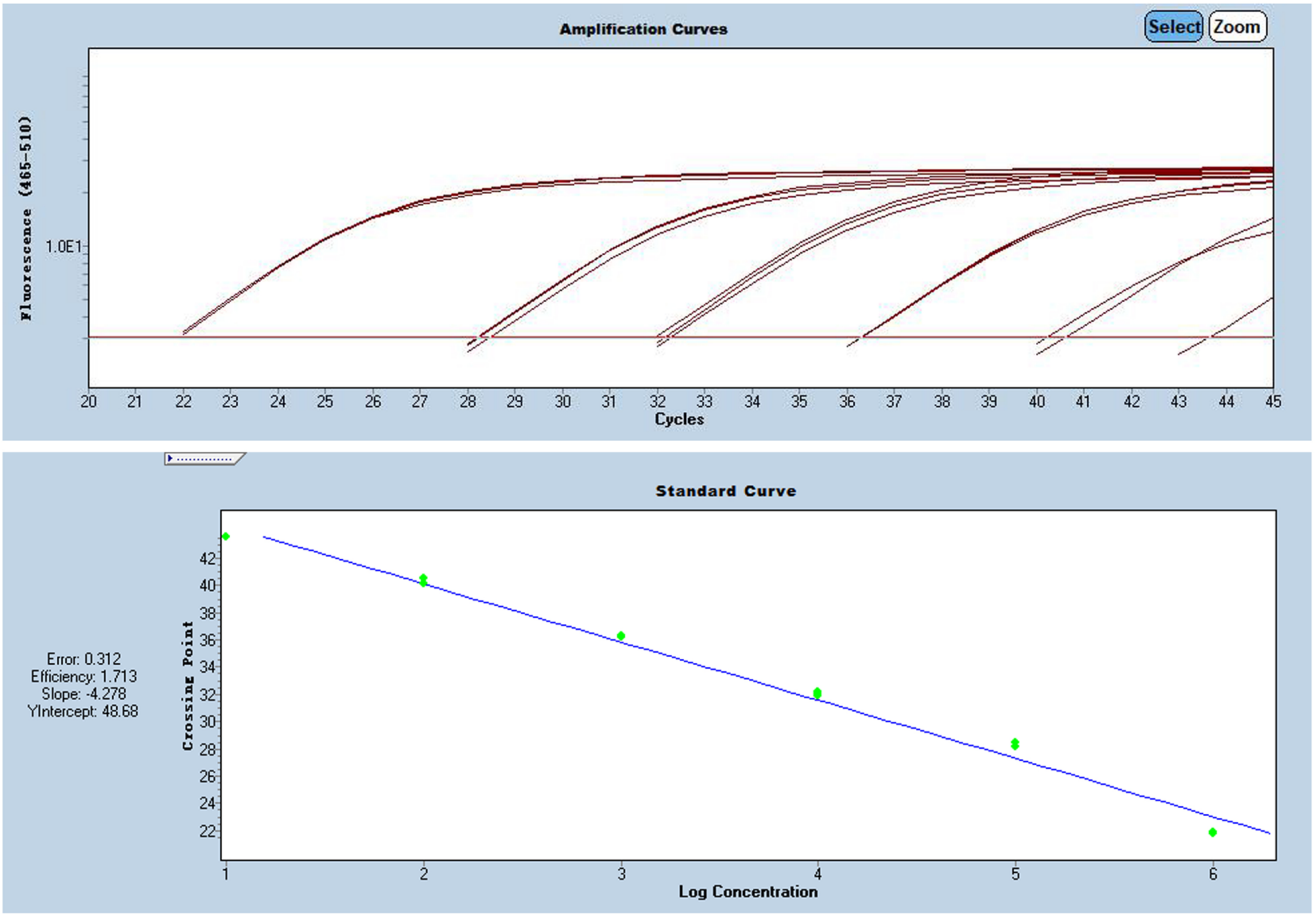
Pre-experiment of RdRP-gene amplification curve

RdRP, N and E-genes were evaluated using serially diluted standards. Results in Additional file 1: Table S1 showing E-gene was more suitable for quantification after SARS-CoV-2 virus infection is confirmed by RdRP-gene and N-gene, as shown in Figs. 1, 2 and 3, and Additional file 1: Table S1.

### RT-PCR for COVID-19 virus detection

LightMix® Modular EAV RNA Extraction Control (580) kit (Roche, Germany. Product No. 07654243001) was used for extraction and reverse-transcription PCR control.

LightCycler® Multiplex RNA Virus Master kit (Roche, Germany. Product No. 06754155001) one-step real-time reverse-transcription PCR kit was used for reverse-transcription RT-PCR.

LightMix® Modular SARS-CoV (COVID19) E-gene kit (Roche, Germany. Product No. 09155368001) based on a previously published assay targeting E gene (envelope protein gene) of coronavirus was employed on Roche cobas® Z480 RT-PCR system [12]. The sequence of forward primer is 5’-ACAGGTACGTTA ATAGTTAATAGCGT-3’ and the reverse primer is 5’-ATATTGCAGCAGTACGCACACA-3’, targeting a 76-bp-long fragment from a conserved region in the E gene with FAM-labeled hydrolysis probes (530 channel) as FAM-ACACTAGCCATCCTTACTGCGCTTCG-BBQ [5].

The assay was according to the kit instruction manual. Primers and probes of target gene and EAV (dry powders) were centrifuged for 2 min at 12000 rpm and pre-dissolved with 50 μL PCR-grade water. Positive control of target gene RNA was centrifuged for 2 min at 12000 rpm and pre-dissolved with 320 μl 10 mM Tris buffer. PCR-grade water is employed as negative control and tested from RNA extraction. One positive control and 3 negative controls were of minimal need for each test.

A 20-μL reaction contained 10 μL of RNA, 0.5 μL of primer/probe premix, 0.5 μL of EAV primer–probe premix, 4.0 μL of RT-PCR Reaction Mix, 0.1 μL of RT-Enzyme Solution and 4.9 μL of PCR-grade water. Thermal cycling was performed on Roche cobas® z480 at 55 °C for 5 min for reverse transcription, followed by 95 °C for 5 min and then 45 cycles of 95 °C for 5 s, 60 °C for 15 s with single acquisition and 72 °C for 15 s, followed by 30 s of 40 °C for cooling down.

### Establishment of the standard curve

The E-gene RNA single positive control (Tib-Molbiol, Germany. Product No. 30743771) contains 10E10 copies of RNA in one vial. 10E7 copies /μL stock solution was obtained by dissolved one vial of E-gene RNA in 1000 µl PCR-grade water. The stock solution was further dispensed into 15 μL/tube and stored in PCR tubes at − 18 to 20 °C to avoid freezing and thawing.

Dilution rows were freshly prepared every day. One tube of stock solution was thawed and dilution rows from 10E6 to 10E0 copies/μL was obtained by serial 1:10 dilution (10 μL of solution diluted with 90 μL of PCR-grade water).

In each experiment, 10 μL of each concentration of dilution rows (from 10E6 to 10E0 copies/μL) in a 20-μL reaction was tested. Standard curve generated with the clinical sample together in each batch was used for viral load calculation.

### RNA extraction of clinical samples

RNA was extracted from clinical samples using the MagNA Pure 96 system (Roche, Germany) with the High Pure Viral RNA Kit (Roche, Germany. Product No. 11858882001).

### Performance evaluation

Limit of detection (LOD), lower limit of quantification (LLOQ) and inter/intra-run variability were evaluated. The limit of detection was determined by analyzing each of 20 replicates of 10E1 copies/μL and 10E0 copies/μL obtained from E-gene RNA dilution rows. The concentration at or above 95% detection probability is accepted as LOD. Lower limit of quantification (LLOQ) was determined by analyzing each of 6 replicates of dilution series from 10E6 to 10E1 copies/μL. The concentration at lower than 15% coefficient of variation (CV) is considered as LLOQ. For estimation of inter/intra-run variability, we analyzed each of 5 positive samples and 5 negative samples confirmed by an EU CE certificated KingDIAN COVID-19 RT-PCR assay kit (DIAN Biotechnology. China) 3 times inter- and intra-run.

## Results

### Performance of the detection

No false positive or false negative results occurred in negative and positive controls in all tests. The cycle threshold (Ct) values of positive controls were between 29 and 31.

In the limit of detection (LOD) determination, for 20 replicates of 10E1 copies/μL, all of the replicates showed positive results with Ct values between 35.2 and 36.7. The coefficient of variation (CV) is 1.03%. For 20 replicates of 10E0 copies/μL, 5 showed undetected results. Therefore, we believe that the actual LOD of the test is between 10E1 and 10E0 copies/μL and the confirmed LOD is 10E1 copies/μL.

**Fig. 4:**
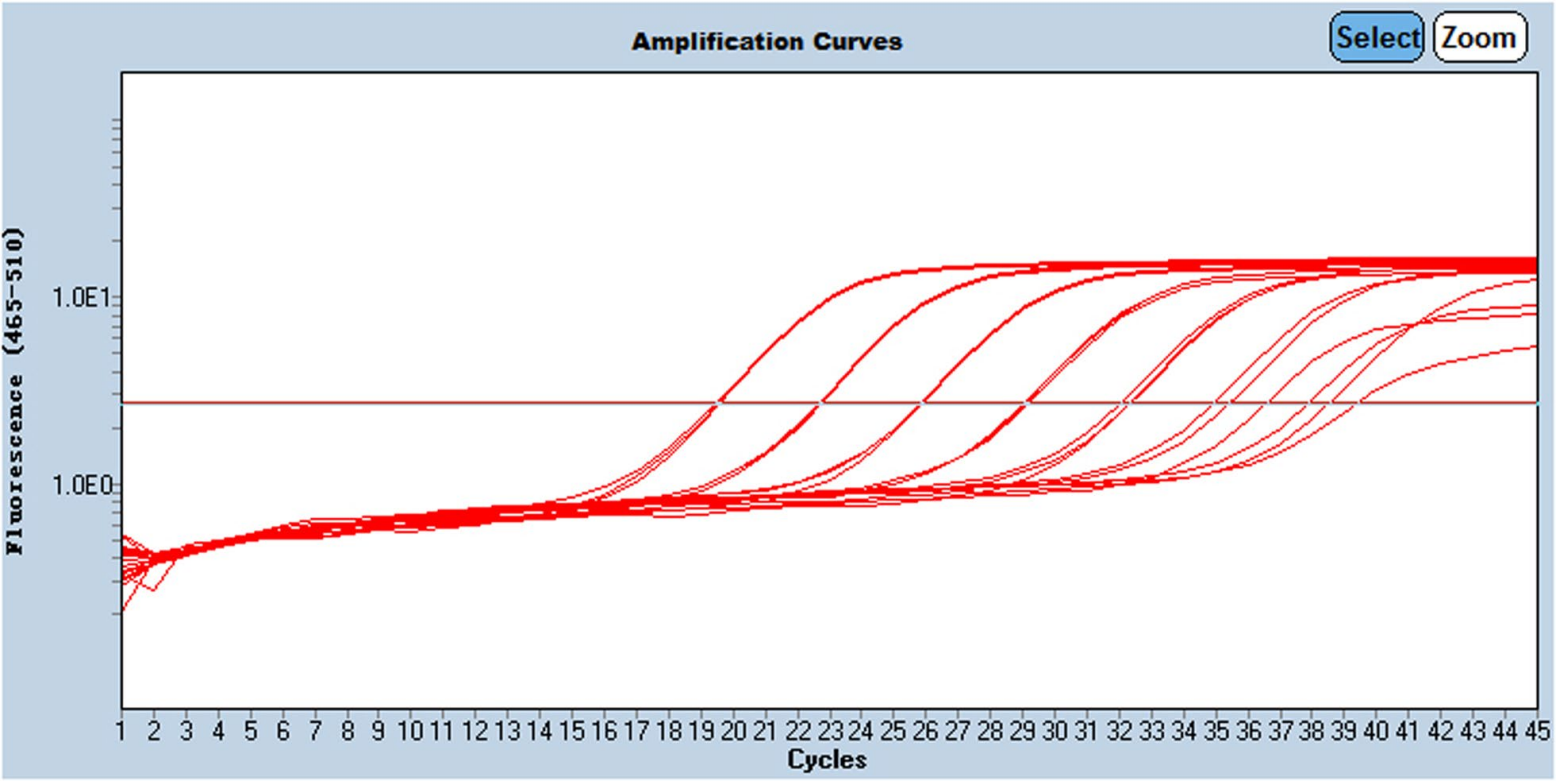
Three replicates inter run of a dilution series from 10E6 to 10E0 copies/μL

For the lower limit of quantification (LLOQ) determination, the CV at 10E2 copies/μL is 11.05% and 50.33% at 10E1 copies/μL. other CV values of higher concentration were 2.45% at 10E6 copies/μL, 2.46% at 10E5 copies/μL,1.36% at 10E4 copies/μL and 5.26% at 10E3 copies/μL. Those results provided an LLOQ of 10E2 copies/μL. So we chose dilution rows from 10E6 to 10E2 copies/μL for the establishment of the standard curve in daily testing, as shown in Fig. 4.

For inter/intra-run variability, all CV values of 5 confirmed positive samples were below 15%, all confirmed negative samples reported undetected.

## Discussion

COVID-19 has become a high risk to the general population and healthcare workers worldwide. Facing the current situation that high infectivity and a large number of infected people, the development of accurate measurement and rapid detection methods is crucial. RT-PCR is widely deployed in the daily clinical assay of virus nucleic acid, which is of great importance for the diagnosis and monitoring of COVID-19. As viral load plays an essential part in diagnostic virology, we developed a quantitative RT-PCR method for COVID-19 virus detection in this study.

LightMix® Modular SARS-CoV (COVID19) E-gene kit was selected to develop the quantitative RT-PCR method. According to the description of the kit and previous report [10], this assay detects SARS and COVID-19 pneumonia virus as well as other bat-associated SARS-related virus (Sarbecovirus), with no cross-reactivity with common human respiratory CoV NL63, 229E, HKU, OC43 or MERS. Since there is no evidence of the prevalence of SARS and other associated SARS-related viruses currently, we chose this kit for the establishment of quantitative detection of the COVID-19 virus.

Several researches have evaluated viral load by Ct values of RT-PCR [13]. However, due to the difference of amplification efficiency and the noise band adjustment, the Ct values of same sample could be significantly different in different tests, especially for low viral load samples. That’s why the standard curve is deployed in most quantitative RT-PCR methods. In this study, we used dilution rows of an RNA standard to establish the standard curve, which enabled us to quantify viral RNA. The performance of the assay is acceptable with a limit of detection (LOD) below 10E1 copies/μL and lower limit of quantification (LLOQ) as 10E2 copies/μL.

There are still some limitations of this assay. The E gene RNA used to establish the standard curve is unstable in solution, especially at low concentration dilution. It could not be used for RNA extraction. Therefore, the viral loads reported by the assay could only reflect the viral loads in extracted RNA solution. Then, considering the current variant of SARS-CoV-2, such as B.1.351 Beta variant or GH501Y.V2 first detected in South Africa in October 2020, and the P.1 variant known as Gamma variant or GR/501Y.V3, which was identified in December 2020 in Brazil and was first detected in the US in January 2021, the detection effect of these mutated viruses will be evaluated in further study.

In summary, we established a quantitative detection method for the COVID-19 virus based on RT-PCR. This study provides a reference idea for the use of conventional qualitative detection PCR kits for quantitative detection, which can perform quantitative detection of new coronavirus nucleic acid at a lower cost level.

## Supplementary Information

**Additional file 1: Table S1.** E-gene standard curve data summary.

## Data Availability

The datasets used or analyzed during the current study are available from the corresponding author on reasonable request.
