# Supplementary material for: Establishment of a quantitative RT-PCR detection of SARS-CoV-2 virus
Source: Eur J Med Res. 2021 Dec 17;26:147. doi: 10.1186/s40001-021-00608-5 (PMC8677905; doi:10.1186/s40001-021-00608-5)
Supplement: Supplementary file 1 — Additional file 1: Table S1. E-gene standard curve data summary. [file 40001_2021_608_MOESM1_ESM.docx]

**Table S1**. E-gene Standard Curve Data Summary

| **Copies/μL** | **Cq Value** | **Actual Conc.** | **Bias** |
| --- | --- | --- | --- |
| 1.00E+06 | 18.69 ± 0.02 | 1.04E+06 | 4.00% |
| 1.00E+05 | 21.91 ± 0.10 | 9.92E+04 | 0.80% |
| 1.00E+04 | 25.03 ± 0.09 | 1.01E+04 | 1.10% |
| 1.00E+03 | 28.15 ± 0.03 | 1.03E+03 | 2.67% |
| 1.00E+02 | 31.64 ± 0.37 | 8.11E+01 | 18.93% |
| 1.00E+01 | 34.57 ± 0.60 | 9.80E+00 | 2.00% |
